# Describing the burden and characteristics of Aspergillus-related chronic lung disease at Imperial College Healthcare Trust: a 10-year retrospective study

**DOI:** 10.1136/bmjresp-2025-003997

**Published:** 2026-06-09

**Authors:** Tim Venkatesan, Navjeet Nagi, Lisa Nwankwo, Darius Armstrong-James, Meg Coleman

**Affiliations:** 1 Imperial College Healthcare NHS Trust, London, England, UK; 2 Pharmacy Department, Royal Brompton Hospital, Guy's and St Thomas’ NHS Foundation Trust, London, England, UK; 3 Department of Infectious Disease Epidemiology, MRC Centre of Global Infectious Disease Analysis, Imperial College London School of Public Health, London, England, UK; 4 Department of Infectious Diseases, Imperial College London, London, UK

**Keywords:** Aspergillus Lung Disease, Asthma, Clinical Epidemiology, Infections

## Abstract

**Background:**

The epidemiology of *Aspergillus*-related chronic lung disease remains poorly defined. We aimed to characterise the local burden at Imperial College Healthcare National Health Service Trust, London, across a 10-year period (2014–2024).

**Methods:**

Electronic health records were reviewed to identify individuals tested for serological markers of *Aspergillus* species infection after which thoracic CT scans were reviewed for features of *Aspergillus*-related chronic lung disease. Patients were classified into diagnostic categories based on definitions provided in the recent British Thoracic Society Clinical Statement.

**Results:**

In total, 334 individuals met criteria for serologic allergic bronchopulmonary aspergillosis (sABPA), 145 for ABPA, 74 for chronic pulmonary aspergillosis (CPA), 38 for simple aspergilloma and 11 with CPA-ABPA overlap. Serological responses varied: ABPA patients had higher median *Aspergillus fumigatus*-specific immunoglobulin (Ig)E titres (7.52 kUA/L, vs 1.94 kUA/L, p<0.05) and a greater proportion were positive for *Aspergillus* antibody (IgG) or precipitins (62.5% vs 32.9%, p<0.05) compared with sABPA. CPA patients had lower median total IgE (94 vs 1494 IU/mL, p<0.05) and *A. fumigatus*-specific IgE (2.24 vs 7.52 kUA/L, p<0.05) than ABPA patients. There was a wide spectrum of radiological presentations across diagnostic categories.

**Conclusions:**

While case numbers are likely underestimated, our findings highlight a substantial and heterogeneous local burden. This improved understanding of local epidemiology will inform our local service development.

**WHAT IS ALREADY KNOWN ON THIS TOPIC**

**WHAT THIS STUDY ADDS**

- There are few studies describing cohorts of patients with *Aspergillus*-related chronic lung disease. This study provides the first detailed description of the burden of *Aspergillus*-related chronic lung disease at a large London National Health Service hospital trust alongside their serological and radiological characteristics.

**HOW THIS STUDY MIGHT AFFECT RESEARCH, PRACTICE OR POLICY**

- Our study highlights the substantial burden of *Aspergillus*-related chronic lung disease and underscores the need to develop and expand the specialist services required to manage them.

## Introduction

*Aspergillus* spp are ubiquitous saprophytic moulds that cause a broad spectrum of chronic lung diseases in susceptible hosts.^1^ These range from allergic, immunoglobulin (Ig)E-mediated disease in patients with asthma to colonisation with progressive destruction of lung parenchyma in individuals with structural lung disease or immunocompromise.^2 3^ Without treatment, this can lead to progressive loss of lung function and even death.^2 4 5^

The global and local epidemiology of *Aspergillus*-related chronic lung disease remains poorly defined.^6^ In the UK, the absence of national surveillance means the true incidence and prevalence of these infections are not known. A modelling study, based on the prevalence of key predisposing conditions, estimated there are between 50 000 cases and 250 000 cases of allergic bronchopulmonary aspeAllergic Bronchopulmonary Aspergillosis (ABPA) and approximately 3600 cases of chronic pulmonary aspergillosisChronic Pulmonary Aspergillosis (CPA) nationally.^7^,^9^ Evidence from UK cohorts remains limited, with the majority of published studies focusing on individuals with cystic fibrosis.^10^,^12^ This uncertainty has impeded the development of specialist services, which are needed given the complexities of both diagnosis and management.^13^

To address the gap in our understanding of local epidemiology, we investigated the burden of *Aspergillus*-related chronic lung infections treated at Imperial College Healthcare National Health Service Trust (ICHT) across a 10-year period (2014–2024). We also examined the demographic, radiological and serological characteristics of affected patients to improve our understanding of our local cohort.

## Materials and methods

We performed a retrospective analysis of patients presenting to ICHT, comprising Charing Cross, Hammersmith and St Mary’s hospitals. Ethical approval was obtained from the NHS Health Research Authority Regional Ethics Committee (REC ref: 24/HRA/4366). Electronic health records were reviewed to identify individuals tested for serological markers of *Aspergillus* species infection between April 2014 and April 2024. These included *Aspergillus* IgG, *Aspergillus* precipitins and species-specific IgE for *A. fumigatus* or *A. niger*. Demographic data (age, sex and ethnicity) were collected for each individual. Test results were classified as positive or negative according to thresholds defined in the recent British Thoracic Society (BTS) Clinical Statement and the revised The International Society for Human and Animal Mycology (ISHAM)-ABPA working group guideline (table 1).^13 14^ For patients with positive serology, total IgE levels and eosinophil counts were obtained.

**Table 1:** Diagnostic criteria for *Aspergillus*-related chronic lung disease.

Category	Definition	
sABPA	1	Total IgE >500 IU/mL and positive *Aspergillus* spp specific IgE (≥0.35 kUA/L)
	2	Absence of radiological requirements for ABPA
ABPA	1	Total IgE >500 IU/mL and positive *Aspergillus* spp specific IgE (≥0.35 kUA/L)
	2	Radiological features: one or more of bronchiectasis, bronchoceles, mucous plugging
CPA	1	Positive *Aspergillus* precipitins or *Aspergillus* antibody (IgG) (≥40.0 mg/L)
	2	Radiological features: one or more of cavity, mycetoma, pleural thickening or lung fibrosis alongside evidence of radiological progression
Simple aspergilloma	1	Mycetoma visible on imaging
	2	No evidence of progression on successive radiology (ie, not CPA)
CPA-ABPA overlap	1	Individually meets criteria for both CPA and ABPA

ABPA, allergic bronchopulmonary aspergillosis; CPA, chronic pulmonary aspergillosis; Ig, immunoglobulin; sABPA, serological allergic bronchopulmonary aspergillosis.

Radiology reports from all thoracic CT scans performed at ICHT during the same time period were reviewed for features of *Aspergillus*-related chronic lung disease.^13 15^ Reports were identified using a structured search strategy, which included both disease nomenclature and characteristic radiological features (eg, bronchiectasis, mucous impaction). The full search terms are provided below.

“ABPA, air crescent sign, allergic bronchopulmonary aspergillosis, aspergiloma, aspergillloma, aspergilloma, aspergillomas, aspegilloma, aspirgilloma, bronchiectasis, CCPA, centrilobular nodules, CFPA, chronic cavitatory pulmonary aspergillosis, chronic fibrosing pulmonary aspergillosis, chronic pulmonary aspergillosis, CPA, halo sign, hyperattenuated mucous sign, intracavitary bodies, intracavitary body, intracavitary mass, intracavitary masses, monod sign, mosaic attenuation, mucous impaction, mycetoma, mycetomas”

Hospital medical record numbers were used to link demographic data, serological results and radiology reports into a single dataset. For individuals with repeat serology, cases were classified as positive if at least one result met diagnostic thresholds. Radiology reports were aligned chronologically and assessed for characteristic features of *Aspergillus-*related chronic lung disease. Patients were classified as positive if features were documented on at least one scan, with a diagnosis of CPA requiring evidence of radiological progression over time. For cases with bronchiectasis, mucous plugging, cavities or mycetomas, the lobar distribution was recorded. Radiologically reported comorbidities or prior pathology (eg, tuberculosis), were extracted if noted in the scan request or report; however, these data were excluded from analysis due to inconsistent documentation. Patients were classified into diagnostic categories based on definitions provided in the BTS Clinical Statement^13^: serological ABPA (sABPA), ABPA, CPA, simple aspergilloma and CPA-ABPA overlap (table 1). We additionally included the categories sABPA, for individuals meeting serological thresholds without documented ABPA-type radiological changes on CT, and CPA-ABPA overlap, for those meeting criteria for both CPA and ABPA (table 1). As per the BTS Clinical Statement and ISHAM-ABPA working group clinical practice guideline, we used a cut-off of >0.5 × 10⁹/L to define an elevated eosinophil count.^14^

Statistical analyses were performed in R Studio. For participants with multiple serological results, individual mean values were calculated, and these per-patient means were summarised by their median within each diagnostic category. Continuous variables were compared using the Mann-Whitney U test and categorical variables with the χ^2^ test. As many *Aspergillus* precipitins or *Aspergillus* antibody (IgG) tests were reported qualitatively, comparative quantitative analysis of median values was not feasible. To investigate potential differences in disease prevalence across ethnic groups, observed ethnic distributions within each diagnostic category were compared with the expected distribution derived from pooled 2021 census data for the boroughs served by ICHT (Hammersmith and Fulham, Westminster, Kensington and Hounslow).^16 17^ χ^2^ goodness-of-fit tests were used to assess deviations from expected proportions, and standardised residuals were calculated to identify ethnic groups that were over-represented or under-represented relative to the local population.

## Results

### Demographics

In total, 334 individuals met criteria for sABPA, 145 for ABPA, 74 for CPA, 38 for simple aspergilloma and 11 with CPA-ABPA overlap. A male predominance was observed across all groups. The mean age was in the sixth decade for all categories except sABPA (39.2 years). When compared with the local population, there was no significant difference in the ethnic distribution for patients with ABPA (χ²=2.83, p=0.42) or simple aspergilloma (χ²=2.62, p=0.45). In contrast, black ethnicity was over-represented among patients with CPA (χ²=12.56, p<0.05, standardised residual (SR)=2.74) with a similar finding seen in individuals with sABPA (χ²=110.25, p<0.05, SR=8.67) (table 2).

**Table 2:** Demographics of patient with *Aspergillus-*related chronic lung disease.

	sABPA	ABPA	CPA	ABPA-CPA overlap	Simple aspergilloma	Local population
Total	334	145	74	11	38	
Male	199 (59.6%)	92 (63.4%)	41 (55.4%)	7 (63.6%)	28 (73.7%)	
Female	135 (40.4%)	53 (36.6%)	33 (44.6%)	4 (36.4%)	10 (26.3%)	
Mean age in years	39.2	64.3	64.3	59.4	62.7	
Ethnicity						
Total	243	123	71	9	32	815 804
White (%, SR)	75 (30.9%, −5.04)	75 (61.0%, 0.93)	42 (59.2%, 0.5)	5 (55.6%)	19 (59.4%, 0.35)	446 888 (54.8%)
Asian (%, SR)	52 (21.4%, −0.08)	22 (17.9%, −0.89)	7 (9.9%, −2.13)	1 (11.1%)	4 (12.5%, −1.11)	176 419 (21.6%)
Black (%, SR)	61 (25.1%, 8.67)	12 (9.8%, 0.4)	13 (18.3%, 2.74)	2 (22.2%)	2 (6.3%, −0.47)	70 998 (8.7%)
Other (%, SR)	55 (22.6%, 3.13)	14 (11.4%, −1.01)	9 (12.7%, −0.48)	9 (11.1%)	7 (21.9%, 1.02)	121 499 (14.0%)
χ^2^ statistic (p value)	110.25 (p<0.05)	2.83 (p=0.42)	12.56 (p<0.05)	/	2.62 (p=0.45)	

Total numbers for the local population were taken from pooled 2021 census data for the boroughs served by ICHT (Hammersmith and Fulham, Westminster, Kensington and Hounslow). χ2 statistic displayed for χ2 goodness of fit test comparing the expected ethnic distribution based on the local population to each disease category. This test was not performed for the ABPA-CPA overlap group due to small patient numbers.

ABPA, aspergillus bronchopulmonary aspergillosis; CPA, chronic pulmonary aspergillosis; sABPA, serologic ABPA; SR, standardised residual.

### Serological results

Serological responses varied between categories. Compared with individuals with sABPA, those with ABPA had statistically higher median *Aspergillus fumigatus*-specific IgE titres (7.52 kUA/L, IQR 1.90–25.60 vs 1.94 kUA/L, IQR 0.76–7.67; Mann-Whitney U=16 483, p<0.05) and among those tested, a significantly greater proportion of individuals were positive for *Aspergillus* antibody (IgG) or precipitins (62.5% vs 32.9%, χ^2^=14.2, p<0.05). Individuals with ABPA were also more likely to have elevated eosinophil counts (50.3% vs 38.0%, χ^2^=5.81, p<0.05). Conversely, the median total IgE for sABPA was higher than that seen in ABPA (2033 IU/mL, IQR 944–4299 vs 1494 IU/mL, IQR 732–3696, Mann-Whitney U=27 777, p value <0.05) (table 3).

**Table 3:** Median serological values for different categories of *Aspergillus-*related chronic lung disease.

		sABPA	ABPA	CPA	Simple aspergilloma	ABPA-CPA overlap
Total		334	145	74	38	11
*Aspergillus* Ab (IgG) or precipitins	No. tested positive (%)	26/79 (32.9)	60/96 (62.5)	74/74 (100)	25/35 (71.4)	11/11 (100)
	% total cohort positive	26/334 (7.8)	60/145 (41.4)	74/74 (100)	25/38 (65.8)	11/11 (100)
*Aspergillus fumigatus*-specific IgE (kUA/L)	No. tested positive (%)	332/333 (99.7)	144/145 (99.3)	33/66 (50.0)	27/36 (75.0)	11/11 (100)
	% total cohort positive	332/334 (99.4)	144/145 (99.3)	33/74 (44.6)	27/38 (71.1)	11/11 (100)
	Median (IQR)	1.94 (0.76–7.67)	7.52 (1.90–25.60)	2.24 (0.99–10.67)	1.08 (0.35–5.27)	5.39 (2.21–30.48)
*A. niger*-specific IgE (kUA/L)	No. tested positive (%)	13/21 (61.9)	14/20 (70.0)	4/21 (19.0)	2/36 (2.9)	1/3 (33.3)
	% total cohort positive	13/334 (3.9)	14/145 (9.7)	4/74 (5.4)	2/38 (5.3)	1/11 (9.9)
	Median (IQR)	0.56 (0.19–1.04)	1.36 (0.31–3.12)	/	/	/
Total IgE (IU/L)	No. tested positive (%)	334/334 (100)	145/145 (100)	17/57 (29.8)	11/27 (40.7)	11/11 (100)
	% total cohort positive	334/334 (100)	145/145 (100)	17/74 (23.0)	11/38 (28.9)	11/11 (100)
	Median (IQR)	2033 (944–4299)	1494 (732–3967)	93.5 (28–650)	251 (106–927)	1313.5 (34.6–667)
Eosinophil counts	No. tested positive (%)	127 (38.0%)	73 (50.3%)	33 (44.6%)	20 (52.6%)	6 (54.5%)

For *A. niger* specific IgE, median values were not calculated for CPA, simple aspergilloma or ABPA-CPA overlap due to small patient numbers (n<5).

ABPA, aspergillus bronchopulmonary aspergillosis; CPA, chronic pulmonary aspergillosis; Ig, immunoglobulin; sABPA, serologic ABPA; SR, standardised residual.

Compared with ABPA, patients with CPA had lower median total IgE (1494 IU/mL, IQR 732–3696 vs 94 IU/mL IQR 28–650, Mann-Whitney U=6843, p value <0.05) and lower median species *A. fumigatus*-specific IgE (7.52 kUA/L, IQR 1.90–25.60 vs 2.24 kUA/L, IQR 0.99–10.67, Mann-Whitney U=1786.5, p value <0.05) when tested. The proportion of patients with elevated eosinophil counts was similar between groups (50.3% vs 44.6%, χ² = 0.65, p=0.42). Serological responses in individuals with simple aspergilloma were comparable to those in CPA. Median total IgE and the proportion of individuals with eosinophilia were similar between the two groups, but both were lower than that seen in ABPA. In total, 71% were positive for *Aspergillus* antibody (IgG) or precipitins (table 3).

### Radiological results

In the ABPA cohort, 92% (134/143) of patients demonstrated radiological evidence of bronchiectasis, with 24.6% (33/134) exhibiting involvement in more than three lobes. No significant difference was observed in distribution between the upper and lower lung zones. Among individuals for whom the bronchiectasis subtype was reported, 72.0% (54/75) had cylindrical, 8.0% (6/75) varicose, 13.3% (10/75) cystic and 12.0% (9/75) tractional bronchiectasis; 8.0% (6/75) had a combination of more than one subtype. The remaining nine individuals without bronchiectasis had other ABPA radiological features. These included mucous plugging, which was present in 51.0% (73/143) of the overall ABPA cohort, and bronchocele formation, identified in 13.3% (19/143) (table 4).

**Table 4:** Radiological features of *Aspergillus*-related chronic lung disease at Imperial College Healthcare Trust.

Radiology	ABPA	CPA	Simple aspergilloma	ABPA-CPA overlap
Bronchiectasis	134 (92.4)	44 (59.5)	17 (44.7)	11 (100)
Bronchocele	19 (13.1)	3 (4.1)	0 (0)	3 (0)
Mucous plugging	73 (50.3)	14 (18.9)	3 (7.9)	5 (45.5)
Lung nodules	53 (36.6)	21 (28.4)	4 (7.9)	5 (45.5)
Mycetoma	7 (4.8)	36 (48.6)	38 (100)	7 (63.6)
Cavity	16 (11.0)	53 (71.6)	38 (100)	8 (72.7)
Pleural thickening	9 (6.2)	21 (28.4)	8 (21.1)	3 (27.3)
Lung fibrosis	15 (10.3)	40 (54.1)	17 (44.7)	3 (27.3)

Percentage total denoted in brackets.

ABPA, aspergillus bronchopulmonary aspergillosis; CPA, chronic pulmonary aspergillosis.

In contrast, patients with CPA displayed greater variation in radiological features. Among individuals with CPA and simple aspergilloma, mycetomas were predominantly located in the upper lobes (69/74, 93.2%), with no apparent difference in laterality. Although, 11 patients met both radiological and serological criteria for ABPA and CPA, a larger subset demonstrated radiological features of both. This included a proportion of patients with ABPA having evidence of cavitation (11%), mycetoma (4.8%), pleural thickening (6.2%) and lung fibrosis (10.3%). Similarly, 59.5% (44/74) of individuals with CPA had bronchiectasis, with 18.9% (14/74) demonstrating mucous plugging (table 4).

## Discussion

*Aspergillus-*related chronic lung diseases are increasingly recognised and represent a heterogeneous group of infections that are challenging to manage and associated with high morbidity.^4 18^ In this study, we have characterised the local epidemiology of these infections at ICHT, a large London NHS trust. We hope this much-needed local epidemiological data begin to fill the huge gap in our current understanding of the national burden of these infections.

### Total burden

Over a 10-year period, we identified large numbers of patients with *Aspergillus*-related chronic lung disease, including nearly 500 cases of serologically or radiologically confirmed ABPA and 112 cases within the CPA spectrum (simple aspergilloma and CPA).

Current estimates of the national burden are derived from a modelling study largely based on the prevalence of ABPA in asthma (reported at 2.5%, range 0.72–3.5%), which estimated approximately 178 000 (50 000–250 000) ABPA cases within the UK.^7 8^ This study used an adult asthma rate in the UK of 16–18.2% based on figures from a World Health Organisation World Health Survey from 2002.^19^ The current true asthma prevalence is debated, and prevalence data vary depending on definition and data sourced. Using a more recent estimate of 9.6%^20^ to our local population of 800 000^17^ would still give an expected ~2000 ABPA cases.

This indicates our study underestimates the true local numbers of ABPA for which there are many potential reasons. It is known that asthmatics at risk of infection are undertested.^21 22^ Milder cases may also remain underdiagnosed or managed outside hospital settings, while some patients may have presented to other NHS trusts.^23^

Modelling the expected local cases of CPA is more complex. Case numbers will depend on regional variations in underlying predisposing conditions and patterns in referral to tertiary care. The national ratio of CPA-to-ABPA cases (3600:178 000) suggests we would expect ~40 CPA cases locally. The higher numbers observed in our cohort may reflect the increased prevalence of pulmonary tuberculosis and sarcoidosis in London, which together account for ~30% of CPA cases.^24^,^26^ Additionally, our numbers of simple aspergilloma cases are also likely an underestimate because our pathway required positive serology before radiological assessment, and as many simple aspergillomas have normal serology,^27^ additional cases would have been missed.

Several other methodological factors may have contributed to both case underestimation and misclassification. Variability in an individual’s serology, which can be influenced by both exacerbations and treatment, could have resulted in false negatives.^27^,^29^ To mitigate this, we classified cases based on a single positive serological result, given most patients underwent repeat testing. Incomplete records and overlapping chronic lung diseases may have further confounded diagnosis. Finally, because we did not collect patient symptom or lung function data from clinic records, we could not confirm obstructive lung disease in ABPA, which is a BTS diagnostic criterion. Nonetheless, both the BTS and international groups such as ISHAM place greater emphasis on serological and radiological markers.^13 14^

### Demographics

In our local cohort, males were consistently more common than females among the cases observed. While prior ABPA cohorts have reported a balanced gender distribution,^30 31^ some have noticed a similar male bias.^22^ In contrast, male predominance in CPA is well established, reflecting the higher prevalence of predisposing conditions such as tuberculosis and chronic obstructive pulmonary disease (COPD).^32^,^35^

Geographic variation in ABPA prevalence and presentation is well recognised,^6 36^ with genetic associations extensively described.^37^,^41^ However, few studies have addressed racial disparities in *Aspergillus*-related chronic lung disease. In the USA, a recent study reported ABPA prevalence was the highest among non-Hispanic black individuals,^22^ which mirrored rates of asthma prevalence in the general population. Whether this finding reflects socioeconomic disparities rather than inherent ethnic risk is not clear. In contrast, UK primary care data found asthma prevalence to be the highest among Caucasian individuals,^42^ and ethnicity was not predictive of ABPA development in a longitudinal cystic fibrosis cohort.^10^ In our cohort, ABPA prevalence by ethnicity mirrored the local population, although the lack of observed difference might be attributable to small patient numbers. The over-representation of black ethnicity in sABPA remains unexplained, and there are limited published data for comparison.

For CPA, global burden reflects the distribution of predisposing conditions such as tuberculosis and sarcoidosis.^43 44^ The over-representation of black ethnicity in our local cohort may reflect higher rates of these conditions.^26 45 46^ Yet, given that tuberculosis incidence is the highest in the UK among individuals of Asian ethnicity, one might have expected a higher incidence of CPA in this group.^26 45 47^

### Serological and radiological characteristics

The serological and radiological features of our cohort were broadly consistent with what has been reported in other cohorts. In ABPA, median total IgE (1494 IU/L, IQR 732–3967) and median *A. fumigatus-*specific IgE (7.52 kUA/L, IQR 1.90–25.60) were comparable to other cohorts.^48 49^ Reported sensitivity of *Aspergillus* precipitins or IgG in ABPA ranges from 27% to 70%,^50^,^53^ and our observed rate (62.5%) fell within this. Serologic ABPA is widely considered a precursor to ABPA with radiological changes.^54 55^ Consistent with this, individuals with radiological ABPA demonstrated higher *A. fumigatus*-specific IgE titres (7.52 kUA/L vs 1.94 kUA/L), a greater frequency of *Aspergillus* antibody (IgG) or precipitins positivity (62.5% vs 32.9%) and more frequent eosinophilia (50.3% vs 38.0%). It is notable, however, that median total IgE was higher in sABPA than ABPA (2033 IU/L vs 1494 IU/L). We suspect this finding may reflect treatment effects in the ABPA group, given both steroids and anti-fungals have been shown to decrease total IgE.^56 57^

Although we have drawn a distinction between simple aspergilloma and CPA being the evidence of radiological progression, these represent a continuum. It is notable that, despite no radiological evidence of progression or localisation invasion, 71.4% of our simple aspergilloma group were serologically positive for IgG or precipitins, which suggests seroconversion may precede radiologically visible progression in some patients. It is established that individuals with CPA often have high serum total IgE and *Aspergillus* spp IgE.^12 20 21^ However, unsurprisingly, we found the median total IgE and *Aspergillus* spp IgE to be higher in individuals with ABPA (1494 IU/L vs 93.5 IU/L, 7.52 kUA/L (1.90–25.60) vs 2.24 kUA/L).

Our cohort also highlights the considerable serological and radiological overlap between ABPA and CPA, with 11 individuals fulfilling diagnostic criteria for both. This phenomenon is well described.^258^,^60^ Modelling studies estimate that 10–15% of ABPA cases progress to CPA.^7 61^ In our cohort, 11% (16/145) of ABPA patients had radiological evidence of cavitation, with fewer showing mycetoma or pleural thickening. It is thought that chronic inflammation in ABPA drives progressive structural damage, leading to cavitation and localised invasion resulting in seroconversion.^2^ The converse is less well described,^44^ although as discussed, we observed widespread T helper 2 cell-mediated IgE responses to *Aspergillus* spp in individuals with CPA.

### Limitations and further work

We acknowledge several limitations to this study. First, we likely underestimate the true case numbers due to undertesting, missing data and cases managed outside the hospital setting. Second, due to feasibility, we did not review individual notes and therefore could not reliably apply clinical diagnostic criteria (eg, asthma/CF status, symptomology, spirometry results) when classifying patients. This would be valuable to explore further. We also did not collect data on patient treatment nor outcomes and having established this local cohort, this will form a logical next route of investigation.

## Concluding remarks

This study characterises *Aspergillus*-related chronic lung disease at a single London NHS trust. While case numbers are likely underestimated, we demonstrate a substantial burden and define the relative proportions of cases managed locally. These findings improve understanding of local epidemiology, with direct implications for service planning and resource allocation. We hope the formation of this local dataset will facilitate further projects looking at local treatment outcomes and antifungal stewardship. As further epidemiological reports emerge, we believe there is a strong case for developing a national registry of ABPA and CPA cases, which would further improve understanding of national disease burden and treatment outcomes, alongside promoting data sharing across treating centres to support capacity building and more efficient specialist services.

## Data Availability

Data are available upon reasonable request.
